# A systematic review of relational-based therapies for the treatment of auditory hallucinations in patients with psychotic disorders

**DOI:** 10.1017/S003329172200143X

**Published:** 2022-08

**Authors:** Laura Dellazizzo, Sabrina Giguère, Nayla Léveillé, Stéphane Potvin, Alexandre Dumais

**Affiliations:** 1 Research center of the Institut Universitaire en Santé Mentale de Montréal, Montreal, Canada; 2 Faculty of Medicine, Université de Montréal, Montreal, Canada; 3 Institut national de psychiatrie légale Philippe-Pinel, Montreal, Canada

**Keywords:** Auditory hallucinations, psychotic disorders, relational therapies, systematic review

## Abstract

**Background:**

Auditory hallucinations in patients with psychotic disorders may be very distressing. Unfortunately, a large proportion of individuals are resistant to pharmacological interventions and the gold-standard cognitive-behavioral therapy for psychosis offers at best modest effects. To improve therapeutic outcomes, several therapies have been created to establish a relationship between voice-hearers and their voices. With increasing literature, we conducted a systematic review of dialogical therapies and examined the evidence behind their efficacy.

**Methods:**

A systematic search was performed in PubMed, PsycINFO, Web of Science, and Google Scholar. Articles were included if they discussed the effects of dialogical interventions for patients with psychotic disorders.

**Results:**

A total of 17 studies were included within this systematic review. Cumulative evidence from various therapies has shown that entering in a dialog with voices is beneficial to patients, even those who are resistant to current pharmacological treatments. Heightened benefits have been mainly observed with Relating Therapy and Avatar Therapy/Virtual Reality assisted Therapy, with evidence generally of moderate quality. Both these interventions have shown large to very large effects on voices and voice-related distress as well as moderate to large magnitude improvements on affective symptoms. Though, cognitive-behavioral therapy for command hallucinations and making sense of voices noted no improvements on voices.

**Conclusions:**

Literature on relational-based interventions with a strong emphasis on the relational aspects of voice hearing has shown positive effects. Results suggest that these dialogical therapies might surpass the efficacy of current gold-standard approaches.

## Introduction

Psychotic disorders, such as schizophrenia, are chronic disorders that have major consequences in the lives of people suffering from the disorder, including increased social isolation, distress and depression (Pješčić et al., 2014) and carries a large economic burden for society (Jin & Mosweu, 2017). Notably, 60% to 80% of patients suffering from schizophrenia spectrum disorders experience auditory hallucinations (AH), commonly referred to as hearing voices (Waters et al., 2014), which have been associated to increased levels of depression and distress (Han et al., 2012). Pharmacological treatments are used as first-line treatment for AH in schizophrenia, yet 30% to 50% of patients are resistant to such an approach (Howes et al., 2017; Meltzer, 1997). Furthermore, beyond side effects on health associated with long term use of antipsychotic medication, this treatment approach does not aid patients to learn to live better and cope with their voices (Howes et al., 2017). Consequently, other therapeutic modalities have emerged to palliate these shortcomings, including psychosocial therapies, with the gold standard being cognitive-behavioral therapy (CBT) (National Collaborating Centre for Mental Health, 2014). However, the efficacy of CBT is only of small to moderate magnitude as evidenced by meta-analyses and smaller effect sizes are found at longer term follow-ups (Avasthi, Sahoo, & Grover, 2020; Burns, Erickson, & Brenner, 2014; Hazell, Hayward, Cavanagh, & Strauss, 2016; Jauhar et al., 2014; Sarin, Wallin, & Widerlov, 2011; Turner, van der Gaag, Karyotaki, & Cuijpers, 2014; van der Gaag, Valmaggia, & Smit, 2014). Consequently, interventions targeted at changing patients' beliefs regarding their voices may not be sufficient to reduce the AH distress, leading to the need for other therapeutic avenues.

Thus, more recent therapeutic models to unfold the voice-hearer to voice relationship have been emerging. These models conceptualize voices within an explicitly interpersonal relational framework since most voice-hearers acknowledge maintaining some sort of a relationship with their voices (Hayward, Strauss, & McCarthy-Jones, 2014; McCarthy-Jones et al., 2014). These approaches are based on developing evidence that maintaining a direct dialog with one's voices can lead to beneficial outcomes including the development of a more constructive relationship with these voices and increase the sense of control (Corstens, Longden, & May, 2012; Deamer & Wilkinson, 2015; Hayward, Berry, & Ashton, 2011; Raffard & Bortolon, 2021; Schnackenberg & Martin, 2014; Thomas et al., 2014). Considering the increased interest in relational therapies for AH in patients with psychotic disorders, we conducted a systematic review to summarize these interventions and their preliminary effects on the primary outcome of interest being AH and on secondary outcomes related to the severity of general symptomatology associated with psychosis.

## Methodology

### Search strategy

A search was independently conducted by two graduate students (L.D. and S.G.) in the electronic databases of PubMed, PsycINFO and Web of Science (from each database's inception date to August 2021). The search string focused on keywords in titles and abstracts. Search terms were chosen to be inclusive of psychotherapies (e.g. ‘Relating therapy’, ‘Talking with voices’, ‘Cognitive behavioral therapy’) and AH (e.g. ‘auditory verbal hallucination’, ‘voices’, ‘voice hearing’). Study designs on individuals principally with psychotic disorders (e.g. schizophrenia spectrum, schizoaffective disorder) were selected. The search syntax was tailored for each database. See online Supplementary Material for the specific search strategy adapted to each database. A secondary search was then conducted in Google Scholar to retrieve gray literature, and reference lists of included manuscripts were screened to ensure at best possible that no pertinent studies were missed. No setting, date or geographical restrictions were applied. Searches were limited to English and French language sources. Authors of articles to which we had restricted access or missing data were contacted.

### Study eligibility

To maximize the number of studies and obtain an overall view on the subject, all study designs, including case reports and case series were included in addition to clinical trials (e.g. randomized controlled trials (RCT)). Studies were included if they were on an intervention that had for goal to target the relationship between voice-hearer and voice and evaluated the effects of the intervention on the primary outcome comprising AH and on symptoms of the illness (i.e. affective symptoms). Studies were excluded if they (i) did not provide a clear definition of their sample or their sample was not mainly on patients with psychotic disorders, (ii) the intervention did not aim to target the relational component towards the voice and was not considered within the branch of relational interventions for AH (e.g. CBT with no relational emphasis during sessions, Acceptance and Commitment Therapy), (iii) comprised of posters, preprints, study protocols with no available data, and non-accessible manuscripts, (iv) discussed the therapeutic components of the therapy without any data on efficacy. To ensure consensus, discussions on the inclusion of studies were held with the research team.

### Data extraction

Data were extracted by L.D. and S.G. using a standardized form. Key information related to the sample, the intervention, the control group, the outcomes measured, and study results were recorded. Effects sizes that were not reported within the article were calculated to help have a magnitude of the effects when possible. The effect sizes were categorized as small (0.2), medium (0.5) and large (>0.8) effects (Cohen, 2013). The details of the studies may be found in online Supplementary Material. Extracted data were independently crosschecked and any queries were resolved by discussion with A.D. and S.P. Furthermore, L.D. and S.G. independently undertook quality assessment using a set of criteria based on the GRADE Checklist (Guyatt et al., 2011) and CONSORT Checklist. Studies were assigned: very low quality, low, moderate-to-low, moderate, moderate-to-high and high (see online Supplementary Material). We assigned higher scores to studies that comprised of single-blind randomized control trial (RCT) with larger sample sizes ideally over 100, compared the treatment to an active control group, and measured their outcome with standardized scales. A similar method has been used in a prior study for quality assessment (Dellazizzo, Potvin, Bahig, & Dumais, 2019). To achieve a high standard of reporting data, the Preferred Reporting Items for Systematic Reviews and Meta-Analyses (PRISMA) guidelines were followed (Moher, Liberati, Tetzlaff, & Altman, 2009).

## Results

### Description of studies

**Fig. 1.**
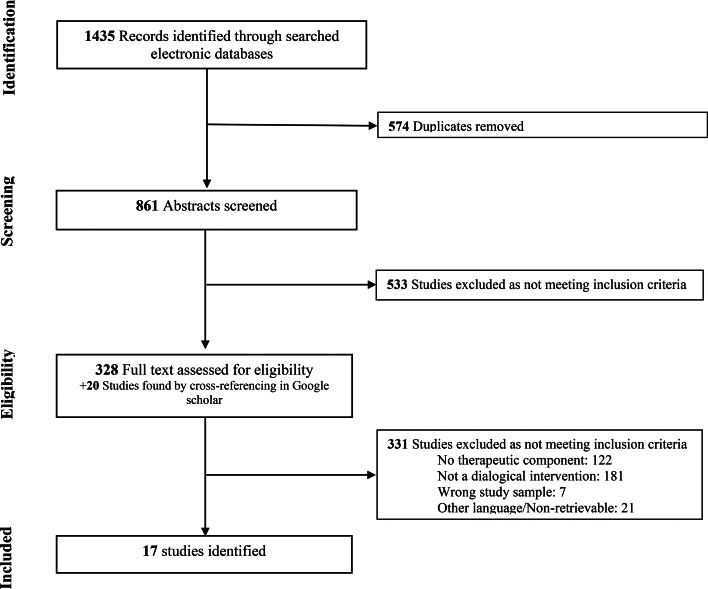
Flow-chart depicting the search strategy to find the studies included in this review.

The systematic search retrieved 861 potential articles that were screened for eligibility after removing duplicates. Amongst these, a total of 17 studies were included within this systematic review, with interventions comprising cognitive-behavioral therapy for command hallucinations, Making sense of voices, Relating Therapy and Avatar Therapy (AT) as well as its variants. The PRISMA flowchart for the inclusion of studies in the systematic review is shown in Fig. 1. Concerning study design, we retrieved 7 case reports/case series, 1 observational qualitative study and 9 clinical trials ranging from very low to high quality of evidence. Refer to online Supplementary Material for a summary of the quality of evidence provided by the included studies. Below we briefly discuss the therapeutic approaches of each relational intervention and examine their effects on the severity of AH and of other symptoms of the illness.

### Cognitive-behavioral therapy for command hallucinations

#### Description

The goal of this therapy is to change the voice-hearer's beliefs that AH have the power over them and that they must obey their request and to reduce harmful compliance behavior. The empty-chair exercise is used where the chair represents a command AH. Voice-hearers begin by sitting in the first chair and describing how they feel living with their voices' omnipotence. When voice-hearers change chair, they are encouraged to put into practice their new beliefs that they learned throughout the therapy sessions such as the beliefs that they must obey the voices' commands or they will be punished, their beliefs on their voices' identity and perception of absolute power. They are also supported to affirm that they will no longer conform to the voices' commands and that they no longer believe the negative comments addressed by the voices. This exercise is also done with the second chair taking the positing of the AH. In this exercise, voice-hearers takes the place of the voice, and they describe how they feel receiving these comments (Birchwood et al., 2014; Meaden, Keen, Aston, Barton, & Bucci, 2013).

#### Outcomes

A RCT (*n* = 197) with evidence graded as being of high quality comparing 25 sessions of cognitive-behavioral therapy for command hallucinations using the empty chair exercise to treatment as usual (TAU) showed that the therapy displayed no significant improvements on the *Psychotic Symptom Rating Scales – AH* (PSYRAT-AH), *Beliefs about voice questionnaire* (BAVQ-R), *Personal Knowledge Questionnaire and Omniscience Scale, Positive and Negative Syndrome Scale* (PANSS), *Calgary Depression scale* (CDS) at post-treatment and at 18-month follow-up (Birchwood et al., 2014).

### Making sense of voices/experience focused counseling

#### Description

Known under various names, such as The Maastricht approach, Talking with voice, Making Sense of Voices, and Experience Focused Counseling (EFC), this approach targets the exploration of prior traumatic events associated to AH and helps voice-hearers accept their voices, make sense of them and learn cope with them (Corstens, Escher, & Romme, 2018; Steel et al., 2019, 2020). The relational component was adapted from the Voice Dialogue, that considers that a person has several selves, with one which is a dissociated part of the self in which a dialog is held (Stone & Stone, 1989). In the therapy, a dialog is held with the most distressing voice to reduce distress. The therapist attempts to resolve the conflict between them and to bring the patient to develop a better understanding of the voice content (Steel et al., 2019). The intervention generally comprises different phases including (i) engagement and psychosocial education, (ii) assessment and formulation, (iii) dialogical work (role-play, two-chair method), (iv) evaluation and consolidation. During the third phase, the therapist asks questions to the voice, who is represented by an empty chair, and voice-hearers may act as an intermediate. These questions aim to determine the origin and reason for the emergence of the voice and thus relate the voice-hearer's life experience to the voices (Steel et al., 2019). The voice is approached less in a dissociative way to the voice-hearer (Corstens et al., 2018). Ultimately, the aim is to bring about a change of attitude with voices, find a positive way to communicate with them and improve the relationship between the voice-hearer and their voices (Corstens et al., 2018, 2012; Steel et al., 2019, 2020).

#### Outcomes

Concerning the effects of the intervention, a case series comprising 15 patients evaluating the effects 20 sessions of Making sense of voices, with evidence evaluated as being of low quality, observed no significant results on the PSYRAT-AH, albeit significant results at follow-up were found on the omnipotence subscale from the BAVQ-R (*d* = 0.78, *p* = 0.02) (Steel et al., 2019). The participants of this case series were invited to participate in a qualitative interview to obtain their opinion on the changes brought by the therapy. Among 12 participants, half reported feeling less distress in relation to the voices and displayed better control over their voices, whereas the others reported either no changes or variations (Steel et al., 2020). Results were measured as being of very low quality.

Furthermore, a pilot RCT, with evidence evaluated as being of low-to-moderate quality, comparing EFC therapy (*n* = 7) to TAU (*n* = 5) showed no within-group nor between-group differences on the PSYRAT-AH at post-treatment (Schnackenberg, Fleming, & Martin, 2017). There were, however, significant effects of EFC on the psychosis sub-scale of the *Brief Psychiatric Rating Scale* (BPRS). Large significant reductions for the therapy on the total BPRS at post-treatment were also observed (*d* = 1.04). Greater improvements were found for EFC over TAU. Though, no significant results were found for the affective subscale.

### Relating therapy/cognitive-behavioral relating therapy

#### Description

Relating Therapy is based on Birtchnell's Relating Theory and Birchwood's interpersonal CBT model of AH (Birchwood et al., 2004; Birtchnell, 2001; Hayward, Jones, Bogen-Johnston, Thomas, & Strauss, 2017). The main goal of Relating Therapy is to reduce the distress felt by the AH by modifying voice-hearers' relationships with their voices and their acquaintances, who are perceived as being harmful. The intervention generally consists of 3 phases: (i) consider the implications of relating to and interacting with voices, (ii) explore themes within the voice-hearers history regarding relating with voices, (iii) explore assertive approaches to relating to and interacting with voices (empty chair exercise, experiential role play). In the third phase, the voice-hearer practices interacting assertively and confidently with the chosen voice whose relationship is problematic in an imaginary way. This can be done using the empty chair exercise, which has been adapted from previous therapies to have a relational component with others. A trialogue is therefore possible between the patient, the therapist and the empty chair representing the voice with whom the voice-hearer desires to improve their relationship. The voice-hearer is encouraged to reflect and consider that how they respond will affect either maintaining or changing the relationship. Also, experiential role play exercises may be used where the therapist and the voice-hearer each play the position of the voice, or the voice-hearer, and they interact using different forms of communication (e.g. passive, aggressive, affirmative). Ultimately, a main goal is to rebalance the equilibrium in the relationship between voice-hearer and voice by diminishing the felt dominance and intrusion of the voice and to help the voice-hearer learn how to interact in an appropriate way in relationships (Deamer & Hayward, 2018; Hayward et al., 2017; Hayward, Overton, Dorey, & Denney, 2009; Paulik, Hayward, & Birchwood, 2013).

#### Outcomes

Concerning therapeutic effects, a case study, with evidence graded as being of very low quality, comprising a patient having followed 12 sessions of Cognitive-behavioral Relating Therapy (a merge between Cognitive therapy for command hallucinations and Relating Therapy) showed post-treatment reductions on the severity of AH and related distress measured with the PSYRAT-AH (Paulik et al., 2013). Though, the perceived power of voices increased following the intervention. Moreover, the authors noted patient improvements on self-esteem measured with the *Rosenberg Self-Esteem Scale* as well as on stress, depression, and anxiety as measured with the *Depression Anxiety and Stress Scale* (DASS) (Paulik et al., 2013). In a case series, with evidence evaluated as being of very low quality, 2 out of 5 patients having followed 12 sessions Relating Therapy showed a reduction on voice distress and 3 reported a better control on voices at post-treatment, and effects were maintained at 3-month follow-up for all but one patient (Hayward et al., 2009). Reductions in negative voice relating were reported by all patients to varying degrees.

This same team conducted a pilot RCT in 2017 comparing Relating Therapy (*n* = 14) to TAU (*n* = 15) and found very large significant improvements at 16 weeks favoring Relating Therapy on the total PSYRAT-AH (*d* = 1.4), more particularly on the distress subscales (*d* = 1.3), which was maintained at the 36-week follow-up (Hayward et al., 2017). Small to moderate between-group effects were noted on the *Voice and you* (VAY) scale, favoring Relating Therapy, albeit non-significant. Evidence was graded as being of moderate quality. Medium-to-large effect sizes on affective symptoms, notably on the depression subscales of the *Hospital Anxiety and Depression Scale* (HADS), were likewise highlighted, with between-group differences favoring Relating Therapy.

### Avatar therapy/virtual reality assisted therapy

#### Description

Although these prior relational based therapies incorporate a dialog with patients' AH, the patient is not in direct and tangible relation with their persecutory voices, and they have to imagine their voices and report their content to the therapist. This shortcoming has led to the development of AT that uses a visual depiction of the AH that enables the therapist to role-play the voice to aid the voice-hearer practice different responses to their experience in a more direct manner (Craig et al., 2018; Leff, Williams, Huckvale, Arbuthnot, & Leff, 2013). The intervention has then been extended by using immersive virtual reality with a head-mounted display to deliver the therapy [Virtual reality assisted Therapy (VRT)] (Dellazizzo, Potvin, Phraxayavong, & Dumais, 2021; du Sert et al., 2018). Briefly, the first session consists of the creation of the avatar, which is personalized according to the description given by the voice-hearers and the voice is transformed by software so that it resembles as closely as possible the one heard by the voice-hearers. In the following sessions, voice-hearers engage in a dialog with their voice animated in real time by the therapist. Notably, the therapist is instructed to begin the therapy with more negativity, mostly repeating what the patient usually hears, and then progress to a more positive and constructive dialog. As an experiential therapy, it primarily focuses on how patients relate to and respond to their voice by addressing emotional regulation, improving self-esteem, and fostering acceptance rather than to directly challenge beliefs about voices. Importantly, this new intervention allows patients to converse with their voice with the aim of improving coping and decreasing distress by addressing power and control within these relationships as well as altering negative relationships and self-perceptions (Beaudoin et al., 2021; Craig, Ward, & Rus-Calafell, 2016; Dellazizzo, Potvin, Phraxayavong, Lalonde, & Dumais, 2018; Leff, Williams, Huckvale, Arbuthnot, & Leff, 2014; Ward et al., 2020).

#### Outcomes

Two small pilot trials (du Sert et al., 2018; Leff et al., 2013) on treatment-resistant patients comparing AT/VRT to TAU showed large reductions at the end of therapy for both trial (*d* = 0.85, *p* < 0.029; *d* = 1.12, *p* < 0.01) and this effect was maintained at the 3-month follow-up (*d* = 1.45, *p* < 0.001; *d* = 1.20, *p* < 0.01), most particularly for the distress subscale (*d* = 1.33, *p* < 0.001 and *d* = 1.31, *p* < 0.001). Both trials observed improvements on beliefs about voices evaluated with the total BAVQ-R scale up to the 3-month follow-up (du Sert et al., 2018; Leff et al., 2013), particularly on malevolence and omnipotence (du Sert et al., 2018). Evidence provided by these pilot trials was graded as being of low-to-moderate quality. These findings on AH were highlighted in a case study as well, with evidence graded as being of very low quality, showing that the patient partner reduced their voices by 80–90% and had better control of voices (Dellazizzo, Percie du Sert, Potvin, Breton, et al., 2018b). Concerning affective symptoms, Leff et al. (2013) showed that, while depressive symptoms as measured with the CDS did not significantly reduce post-treatment, by the 3-month follow-up, there was a significant improvement from baseline. Accordingly, du Sert et al. (2018) showed that VRT produced significant moderate improvements in depressive symptoms measured with the *Beck Depression Inventory* (BDI) both at post-treatment and at follow-up as well as reductions in general symptoms as measured with the PANSS scale (post-treatment: *d* = 0.61, *p* < 0.05; follow-up: *d* = 1.13, *p* < 0.01).

Following, a larger RCT was conducted by Craig et al. (2018) in 150 treatment-resistant patients comparing 6 sessions of AT to supportive counseling. The later intervention comprised a manual-based, face-to-face supportive counseling approach adapted to facilitate the exploration of issues of importance to voice-hearers' lives. Evidence was graded as being of high quality. Between-group differences showed a large effect size in favor of AT on the total PSYRAT-AH scale (*d* = 0.8, *p* = 0.0093). The effects of AT were maintained at the 24-week follow-up. There were significant improvements on the total *Voice Power Differential Scale* (VPDS) (*p* = 0.026) and the acceptation as well as action subscales of the *Voices Acceptance and Action Scale* (VAAS) (*p* = 0.033), which were not maintained at follow-up (Craig et al., 2018). Significant reductions on the total BAVQ-R scale (*p* = 0.018), particularly for omnipotence (*p* = 0.038) were observed, but not maintained at follow-up. Moreover, there were no significant improvements on stress, depression, and anxiety as measured with the DASS and CDS, nor on self-esteem measured with the *Rosenberg Self-Esteem Scale* and positive and negative symptoms evaluated with the scale for *Assessment of Positive and Negative Symptoms* (SAPS and SANS).

Lastly, a one-year randomized trial in 74 treatment-resistant patients comparing 9 sessions of VRT to the gold-standard CBT, which involved of a succession of learning modules and suggested task assignments, showed that VRT produced larger effects on the total PSYRAT-AH (*d* = 1.080, *p* < 0.001) at short-term follow-up, particularly for the distress subscale (*d* = 0.998, *p* < 0.001), and a moderate size effect for the frequency (*d* = 0.701, *p* = 0.021) and attribution scales (*d* = 0.665, *p* = 0.004), which were maintained at 12-month follow-up (Dellazizzo et al., 2021). VRT showed significant improvements from baseline to three-month follow-up on persecutory beliefs (*d* = 0.438). Evidence was graded as being of moderate quality. Furthermore, the effect of VRT was of moderate magnitude (*d* = 0.651) for overall symptomatology and was found to be larger for the affective symptoms (*d* = 0.724 for excited/hostility symptoms and *d* = 0.786 for anxio-depressive symptoms), which was maintained at 12-month follow-up. There was also one statistically significant between-group effect for the anxio-depressive subscale of the PANSS, yielding to a superiority of VRT over CBT (*p* = 0.025).

### Variants of the approach

This innovative therapy has had several variants including its merge with CBT (Dellazizzo, Potvin, Phraxayavong, & Dumais, 2020; Stefaniak, Sorokosz, Janicki, & Wciórka, 2017) and the use of a paper mask (Cichocki, Palka, Leff, & Cechnicki, 2016).

First, a case study was conducted to evaluate the benefits of a less costly version of AT using a mask representing the AH made of Papier-mâché worn by the therapist. At 1-year follow-up, the treatment-resistant patient had considerably reduced their medication, was in remission of positive symptoms, and their AH, when present, did not disrupt their social functioning (Cichocki et al., 2016). Evidence was measured as being of very low quality.

The second variant consisted of investigating the benefits of following CBT and VRT sequentially, which was measured with a case study (Dellazizzo, Potvin, Phraxayavong, Lalonde, et al., 2018) and a proof-of-concept study (Dellazizzo et al., 2020). The case study of an ultra-resistant patient showed that at post treatment the voice worked on during therapy had completely ceased, and this effect was maintained at the 3-month follow-up. The patient also showed a 24% reduction on the total PANSS score (27% for the positive symptoms, 29% for the negative symptoms and 20% for the general symptoms) (Dellazizzo, Potvin, Phraxayavong, Lalonde, et al., 2018). Evidence was evaluated as being of very low-quality. The proof-of-concept (Dellazizzo et al., 2020) with evidence being graded as low quality showed that effects were significant between baseline CBT and 3-month follow-up of VRT for all AH outcomes. The effects of CBT + VRT on AH were of large magnitude (PSYRATS-AH-Total score *d* = 1.043; PSYRATS-AH-Distress *d* = 0.898; PSYRATS-AH-Frequency *d* = 0.859; PSYRATS-AH-Attribution *d* = 1.020; PSYRATS-AH-Loudness *d* = 0.946). Beliefs about voices then increased towards baseline value at follow-up VRT. As for depressive symptoms, the merged therapies showed a significant diminishment of large magnitude between baseline CBT to post VRT (*d* = 0.783, *p* = 0.004) and follow-up VRT (*d* = 1.020, *p* < 0.001). The effect size for overall symptoms from baseline CBT to post VRT was of large magnitude (*d* = 0.953). There were also significant effects of CBT + VRT throughout time points for disorganized (*d* = 1.040) and between baseline CBT to post VRT for excited/hostility symptoms (*d* = 0.667) as well as positive symptoms (*d* = 1.128).

Lastly, the final variant was an intervention combining both CBT and AT approaches as one sole therapy, which was investigated with a case study (Stefaniak et al., 2017) followed by a pilot trial (Stefaniak, Sorokosz, Janicki, & Wciórka, 2019). One of the differences with AT was that during sessions, the therapist is seated next to the patient in the same room. The first sessions are centered on CBT components (e.g. beliefs towards the voices, maladaptive schemes), to let place later to the major component of AT, that is the interaction with the avatar. The case study, with evidence graded as being of very low quality, showed a significant reduction in voice frequency and intrusiveness at the end of 20 therapy sessions, which was maintained at 6 months follow-up. Moreover, the patient felt more capable of facing voices when present (Stefaniak et al., 2017). This same team conducted a pilot study including 23 treatment-resistant patients having followed a shorter version of the therapy (8 sessions), with evidence being evaluated as low quality. There were very large improvements observed in the total PSYRAT-AH at post-treatment (*d* = 1.486, *p* < 0.0001) as well as at 3 months follow-up (*d* = 1.837, *p* = 0.013). This was particularly the case for the negative content and voice distress subscales. Large improvements were also observed on the total VPD at post-treatment (*d* = 1.338, *p* < 0.0001), which was not maintained at follow-up (Stefaniak et al., 2019).

## Discussion

This systematic review aimed to summarize the current state of evidence on relational interventions for psychotic disorders by evaluating the current data provided by case reports and trials. In summary, cognitive-behavioral therapy for command hallucinations and Making sense of voices noted no improvements on voices. On the other hand, Relating Therapy and AT/VRT showed the largest improvements on AH severity, particularly related to voice distress, which were maintained at longer-term follow-up. Both interventions also generated moderate to large effects on affective symptoms. Notably, the results of AT/VRT were confirmed by a few trials provided by 2 independent teams comparing AT/VRT not only to TAU, but also supportive counseling and CBT. Evidence from these trials was evaluated as ranging from low-to-moderate to high quality. Variants of AT/VRT, mainly its merge with CBT suggest larger improvements.

Notably, the results of these relational based interventions may go beyond the small-to-moderate effects that may be obtained with the gold-standard guideline-recommended CBT for psychosis as showcased with several meta-analyses (Avasthi et al., 2020; Burns et al., 2014; Hazell et al., 2016; Jauhar et al., 2014; Sarin et al., 2011; Turner et al., 2014; van der Gaag et al., 2014). Nowadays, there is consequently an increasing tendency for CBT-type interventions to focus less on reassessing beliefs about voices and to begin employing supplementary therapeutic methods to highlight different components of schema, ways of relating to oneself, emotional regulation and interpersonal relationships as observed in the therapies included within this review (Tai & Turkington, 2009). Furthermore, generic CBT may not be amply experiential as an intervention to allow substantial outcome change. Including experiential elements entrenched within the therapy may not only facilitate cognitive and emotional change for patients, but it is also likely to be useful to allow a positive change in relating to voices and other people (Hayward et al., 2014). Instead of trying to challenge beliefs about voices and learn to resist them, Relating Therapy and AT/VRT primarily focuses on how patients interact with their voices by working on improving self-esteem, self-acceptance and emotion regulation. Within such approach, the patient's relationship with their voice is fundamentally viewed in the context of their current and previous significant relationships (Birchwood et al., 2004; Birchwood, Meaden, Trower, Gilbert, & Plaistow, 2000). Beyond the empty chair exercise and role-plays with the therapist used in therapies with positive outcomes, such as Relating Therapy, new technological developments allow to take such exercises to new heights. The use of virtual reality as in AT/VRT is especially valuable in the case of schizophrenia for the treatment of AH since simple exposure is difficult due to the invisibility of voices. AT/VRT creates tangible and experiential dialogical exposure-based experiences to elicit greater feelings of presence in sessions and builds strong emotions while conversing with a personified version of their voices. This method distinguishes AT/VRT from other dialogical approaches in which the therapist must play the role of a facilitator mediating the conversation (i.e. Hayward et al., 2017). The exposure to an avatar of patients' embodied voice is expected to be a distinctive and vigorous tool to reduce fear and distress associated with persecutory voices. Additionally, visualizing the voice may facilitate the process of validating the voice-hearing experience and may modify the flow of dialog with voices through sessions while altering the voice-hearer relationship (Pradhan, Pinninti, & Rathod, 2016). AT/VRT is the latest innovative relational-based therapy combining many elements from previous dialogical therapies to improve treatment efficacy. In doing so, AT/VRT may touch a range of therapeutic targets as shown in several studies (Beaudoin et al., 2021; Dellazizzo, Percie du Sert, Phraxayavong, Potvin, et al., 2018a; Ward et al., 2020) that are relevant to the voice-hearing experience and allow patients to live their experience in a secure therapeutic environment, thus enabling learnings to be more readily transferred to the real world, which may likewise explain the significant improvement observed on subjective quality of life.

Nevertheless, literature on the subject is still in its infancy and more research is necessary in the field of psychiatry to establish high-quality evidence with the use of gold-standard evidence from well-designed single-blind RCTs comprising large samples. Hence, it is worth noting that the quality of evidence was evaluated as being quite variable, ranging from very low quality provided from several case reports to high quality provided from a single-blind RCT. Several reasons account for the lower quality of evidence amongst trials, such as the lack of blinding, of large sample sizes leading to paucity of statistical power, of active control group, and of long-term follow-up. This remains of importance because an RCT with methodological issues is insufficient to create evidence-based practice. Thus, the quality of the studies should be considered to understand the efficacy of interventions. Another limitation is that there were heterogeneities between study samples with a few studies including, albeit at a smaller proportion, individuals with non-psychotic disorders (i.e. personality disorders, mood disorders). Though, it is worth noting that several similarities have been found between voice hearers across diagnoses, which may likewise suggest the transdiagnostic potential of relational-based interventions. Moreover, this review was limited to English- and French-language, peer-reviewed literature published until August 2021. Although we employed a rigorous search strategy, it is possible that relevant literature was excluded in terms of language and timeframe. We nevertheless attempted to retrieve gray literature by conducting a secondary search in Google Scholar and reference lists of included manuscripts were screened to ensure at the best possible that no pertinent studies were missed. Authors of studies in which the approach remained ambiguous concerning the presence or lack thereof a relational component were additionally contacted to obtain additional information on their approaches and studies.

## Conclusion

To conclude, literature on the efficacy of relational-based interventions with a strong emphasis on the relational aspects of voice hearing have shown positive effects. This is notably the case for Relating Therapy and AT/VRT. Results suggest that these dialogical therapies might surpass the efficacy of current gold-standard approaches, such as CBT, that rely heavily on changing voice appraisal. While there is no sole effective intervention that benefits all patients, these dialogical therapies highlight the future of patient-tailored approaches that incorporates several processes (i.e. self-experience, emotion regulation) relevant to possibly enhance the efficacy of standard CBT for voices. These therapies provide opportunities to go over and beyond traditional interventions, whilst allowing therapists to tailor approaches to each individual beyond a one-size fits all method, thereby possibly improving efficacy and the maintenance of skills. With the growth of personalized medicine, future research should be encouraged to achieve a better understanding of factors that may play a role in outcomes and help explain different effects from usual treatment.

## Data Availability

No datasets were generated or analyzed for the current study.

## Supplementary material

For supplementary material accompanying this paper visit https://doi.org/10.1017/S003329172200143X. click here to view supplementary material
