# Supplementary material for: A systematic review of relational-based therapies for the treatment of auditory hallucinations in patients with psychotic disorders
Source: Psychol Med. 2022 Jul 20;52(11):2001–8. doi: 10.1017/S003329172200143X (PMC9386435; doi:10.1017/S003329172200143X)
Supplement: Supplementary file 1 [file S003329172200143Xsup001.docx]

**Supplementary Online Content**

Dellazizzo, Giguère, Léveillé, Potvin & Dumais. A systematic review of relational-based therapies for the treatment of auditory hallucinations in patients with psychotic disorders

**Table S1.** Electronic search strategy for the meta-review conducted.

**Table S2.** Details of the retrieved studies included.

**Table S3.** PRISMA Checklist.

**Table S1.** Electronic search strategy for the meta-review conducted.

| **Database; Search** | **Search terms** |
| --- | --- |
| 1. **PubMed**;   k=695 | (“Cognitive therapy for command hallucinations”[Title/Abstract] OR CTCH[Title/Abstract] OR “Treatment of Resistant Command Hallucinations”[Title/Abstract] OR TORCH[Title/Abstract] OR “Cognitive behavioral therapy”[Title/Abstract] OR CBT[Title/Abstract] OR “Cognitive behavioural therapy”[Title/Abstract] OR CBT[Title/Abstract] OR CBTp[Title/Abstract] OR “Acceptance and commitment therapy”[Title/Abstract] OR ACT[Title/Abstract] OR Mindfulness[Title/Abstract] OR “Metacognitive Interpersonal Therapy”[Title/Abstract] OR MIT[Title/Abstract] OR “Competitive Memory Training”[Title/Abstract] OR COMET[Title/Abstract] OR “Compassionate mind training”[Title/Abstract] OR CMT[Title/Abstract] OR “Cognitive behavioural relating therapy”[Title/Abstract] OR CBRT[Title/Abstract] OR “Schema therapy”[Title/Abstract] OR “Person-based cognitive therapy”[Title/Abstract] OR PBCT[Title/Abstract] OR “Relating therapy”[Title/Abstract] OR RT[Title/Abstract] OR “Dialogical therapy”[Title/Abstract] OR “Talking with voices”[Title/Abstract] OR “Voice dialogue”[Title/Abstract] OR “Virtual reality”[Title/Abstract] OR “Avatar Therapy”[Title/Abstract] OR AT[Title/Abstract] OR “Relational-based”[Title/Abstract] OR “Relational therapies”[Title/Abstract] OR “Dialogical engagement”[Title/Abstract])  AND (“Distressing voices”[Title/Abstract] OR “Command hallucinations”[Title/Abstract] OR “voices”[Title/Abstract] OR “Auditory hallucination”[Title/Abstract] OR “Auditory hallucinations”[Title/Abstract] OR “Auditory verbal hallucination”[Title/Abstract] OR “Auditory verbal hallucinations”[Title/Abstract] OR “Hearing voices”[Title/Abstract] OR “Voice hearing”[Title/Abstract]) |
| 1. **PsycINFO**;   k=264 | (Title:(((“Cognitive therapy for command hallucinations”) OR (CTCH) OR (“Treatment of Resistant Command Hallucinations”) OR (TORCH) OR (“Cognitive behavioral therapy”) OR (CBT) OR (“Cognitive behavioural therapy”) OR (CBT) OR (CBTp) OR (“Acceptance and commitment therapy”) OR (ACT) OR (Mindfulness) OR (“Metacognitive Interpersonal Therapy”) OR (MIT) OR (“Competitive Memory Training”) OR (COMET) OR (“Compassionate mind training”) OR (CMT) OR (“Cognitive behavioural relating therapy”) OR (CBRT) OR (“Schema therapy”) OR (“Person-based cognitive therapy”) OR (PBCT) OR (“Relating therapy”) OR (RT) OR (“Dialogical therapy”) OR (“Talking with voices”) OR (“Voice dialogue”) OR (“voice engagement”) OR (“Virtual reality”) OR (“Avatar Therapy”) OR (“Relational-based”) OR (“Relational therapies”)) AND ((“Distressing voices”) OR (“Command hallucinations”) OR (“Auditory hallucination”) OR (“Auditory hallucinations”) OR (“Auditory verbal hallucination”) OR (“Auditory verbal hallucinations”) OR (“Hearing voices”) OR (“Voice hearing”)))) OR (Abstract:(((“Cognitive therapy for command hallucinations”) OR (CTCH) OR (“Treatment of Resistant Command Hallucinations”) OR (TORCH) OR (“Cognitive behavioral therapy”) OR (CBT) OR (“Cognitive behavioural therapy”) OR (CBT) OR (CBTp) OR (“Acceptance and commitment therapy”) OR (ACT) OR (Mindfulness) OR (“Metacognitive Interpersonal Therapy”) OR (MIT) OR (“Competitive Memory Training”) OR (COMET) OR (“Compassionate mind training”) OR (CMT) OR (“Cognitive behavioural relating therapy”) OR (CBRT) OR (“Schema therapy”) OR (“Person-based cognitive therapy”) OR (PBCT) OR (“Relating therapy”) OR (RT) OR (“Dialogical therapy”) OR (“Talking with voices”) OR (“Voice dialogue”) OR (“voice engagement”) OR (“Virtual reality”) OR (“Avatar Therapy”) OR (“Relational-based”) OR (“Relational therapies”)) AND ((“Distressing voices”) OR (“Command hallucinations”) OR (“Auditory hallucination”) OR (“Auditory hallucinations”) OR (“Auditory verbal hallucination”) OR (“Auditory verbal hallucinations”) OR (“Hearing voices”) OR (“Voice hearing”)))) |
| 1. **Web of Science**;   k=476 | (TS=(“Cognitive therapy for command hallucinations” OR CTCH OR “Treatment of Resistant Command Hallucinations” OR TORCH OR “Cognitive behavioral therapy” OR CBT OR “Cognitive behavioural therapy” OR CBT OR CBTp OR “Acceptance and commitment therapy” OR ACT OR Mindfulness OR “Metacognitive Interpersonal Therapy” OR MIT OR “Competitive Memory Training” OR COMET OR “Compassionate mind training” OR CMT OR “Cognitive behavioural relating therapy” OR CBRT OR “Schema therapy” OR “Person-based cognitive therapy” OR PBCT OR “Relating therapy” OR RT OR “Dialogical therapy” OR “Talking with voices” OR “Voice dialogue” OR “voice engagement” OR “Virtual reality” OR “Avatar Therapy” OR “Relational-based” OR “Relational therapies”))  AND (TS=(“Distressing voices” OR “Command hallucinations” OR “Auditory hallucination” OR “Auditory hallucinations” OR “Auditory verbal hallucination” OR “Auditory verbal hallucinations” OR “Hearing voices” OR “Voice hearing”)) |

^Note.^ A search in Google Scholar with the same keywords and cross-referencing enabled in the finding of an additional 20 articles.

**Table S2. Details of the retrieved studies.**

| **Author, Year** | **Participants** | | **Type of study** | **Control group** | **Number of sessions** | **Results** | **Quality of evidence** |
| --- | --- | --- | --- | --- | --- | --- | --- |
|  | **Total sample size** | **Sample** |  |  |  |  |  |
| *Cognitive behavioral therapy for command hallucinations* | | | | | | | |
| Birchwood et al., 2014 | 197  (98 CTCH,  99 TAU) | SCZ, SZA or mood disorders, had a history of harmful command hallucinations for at least 6 months with recent | Single blind RCT | TAU | 25 | Between group  **Severity of AH**  PSYRAT AH total  Post: NS; 18 months follow-up: NS  PSYRAT AH distress  Post: NS; 18 months follow-up: NS  PSYRAT AH frequency  Post: NS; 18 months follow-up: NS  PSYRAT AH negative content  Post: NS; 18 months follow-up: NS  VPD total  Post: NS; 18 months follow-up: NS  VDP power  Post: NS; 18 months follow-up: NS  **Belief about voices**  BAVQ-R malevolence  Post: NS; 18 months follow-up: NS  BAVQ-R benevolence  Post: NS; 18 months follow-up: NS  BAVQ-R omnipotence  Post: NS; 18 months follow-up: NS  BAVQ-R emotional resistance  Post: NS; 18 months follow-up: NS  BAVQ-R behavioural resistance  Post: NS; 18 months follow-up: NS  BAVQ-R total engagement  Post: NS; 18 months follow-up: NS  BAVQ-R behavioural engagement  Post: NS; 18 months follow-up: NS  Personal Knowledge Questionnaire and Omniscience Scale  Post: NS; 18 months follow-up: NS  **Severity of other symptoms**  PANSS AH  Post: NS; 18 months follow-up: NS  PANS delusion  Post: NS; 18 months follow-up: NS  PANSS positive  Post: NS; 18 months follow-up: NS  PANSS negative  Post: NS; 18 months follow-up: NS  PANSS general  Post: NS; 18 months follow-up: NS  Calgary depression scale  Post: NS; 18 months follow-up: NS | High |
| *Making sense of voices /Experience Focused Counselling* | | | | | | | |
| Steel et al., 2019 | 15 | SCZ, SZA, psychosis NOS, emotionally unstable personality disorder (2), depression (1) | Case series | None | 20 | ***Severity of AH***  PSYRAT AH total  Post: NS; 3 months follow-up: NS  ***Belief about voices***  BAVQ-R malevolence  Post: NS; 3 months follow-up: NS  BAVQ-R benevolence  Post: NS; 3 months follow-up: NS  BAVQ-R omnipotence  Post: NS; **3 months follow-up: d=0.78, p=0.02**  ***Severity of other symptoms***  GAD7  Post: NS; 3 months follow-up: NS | Low |
| Steel et al., 2020 | 12 | SCZ, SZA, psychosis NOS, emotionally unstable personality disorder (2), depression (1) | Observational qualitative | None | 20 | ***Severity of AH***  Qualitative distress  6 reported feeling less distressed by their voice hearing experience than before the intervention started. 3 said their distress had remained the same, 2 said that their distress varied and one reported more distress.  Qualitative control  6 participants reported feeling more in control of their voices, 4 stated that there had been no change in control and 2 stated that their control varied | Very low |
| Schnackenberg et al., 2016 | 12  (7 EFC,  5 TAU) | SCZ, SZA | Nonblind randomised controlled pilot study | TAU | 44 | Within group  ***Severity of AH***  PSYRAT AH total  Post: NS  BPRS-E psychosis  **Post : d=0.91, p=0.025**  ***Severity of other symptoms***  BPRS-E total  **Post: d=1.04, p=0.023**  BPRS Anxiety / depression  Post: NS  Between group  ***Severity of AH***  PSYRAT AH total  Post: NS  BPRS-E psychosis  **Post : d=1.6, CI=0.24; 2.93**  ***Severity of other symptoms***  BPRS-E total  **Post : d=1.3, CI=0.04; 2.61**  BPRS-E Anxiety / depression  Post: NS | Low-moderate |
| *Cognitive behavioural relating therapy* | | | | | | | |
| Paulik et al., 2013 | 1 | SCZ | Case report | None | 12 | ***Severity of AH***  PSYRAT AH total  Post: ↓ 33.33 %*  PRYRAT AH severity  Post: ↓ 28.5 %*  PSYTAT AH distress  Post: ↓ 71.5 %*  VPD  Post: ↑ 36 %*  ***Severity of other symptoms***  DASS depression  Post: ↓ 25 %*  DASS anxiety  Post: ↓ 100 %*  DASS stress  Post: ↓ 90 %*  ***Self-esteem***  RSES  Post : ↑ 100 %* | Very low |

| **Author, Years** | **Participants** | | **Type of study** | **Control group** | **Number of sessions** | **Results** | **Quality of evidence** |
| --- | --- | --- | --- | --- | --- | --- | --- |
|  | **Total sample size** | **Sample** |  |  |  |  |  |
| *Relating therapy* | | | | | | | |
| Hayward et al., 2009 | 5 | SCZ, depression with psychotic features (1) | Case serie | None | 12 | ***Severity of AH***  PSYRAT AH intensity of distress and controllability  Enhanced controllability was reported by 3, while reductions in distress were reported by 2 participants. 1 reported no sustainable change in distress and controllability.  ***Relation with AH***  VAY voice dominance  1-months follow-up: ↓52.4 %, ↑16.7 %, ↓52.7 %, ↓42.9 %, ↓14.3 %; 3-months follow-up: ↓61.9 %, ↑16.7 %, ↓47.3 %, ↓38 %, NA (loss to follow-up)  VAY voice intrusive  1-months follow-up: ↓46,15%, 0%, ↓12,5 %, ↓50%, ↓5,6%; 3-months follow-up: ↓61,54%, ↑50 %, ↓12,5 %, ↓50 %, NA (loss to follow-up)  VAY hearer dependence  1-months follow-up: ↓42.9 %, ↑500 %, ↓ 100 %, ↓58.4 %, ↓77.8 %; 3-months follow-up: ↓85.7 %, ↓ 100 %, ↓ 100 %, ↓58.4 %, NA(loss to follow-up)  VAY hearer distance  1-months follow-up: ↓44.9 %, ↓16.7 %, ↓52.7 %, ↓52.4 %, ↓9.5 %; 3-months follow-up: ↑9.2 %, ↓16.7 %, ↓ 42.2 %, ↓52.4 %, NA (loss to follow-up) | Very low |
| Hayward et al., 2017 | 29  (14 RT,  15 TAU) | SCZ spectrum, BPD (5), depression (4), obsessive compulsive disorder (1), drug-induced psychosis (1) | Blind randomised controlled pilot study | TAU | 16 | Within group  ***Severity of AH***  PSYRAT AH total  Post: d=0.864*, p:NS; 36 weeks follow-up: d=1.049*, p:NS  PSYRAT AH distress  Post: d=0.757*, p:NS; 36 weeks follow-up: d=0.93*, p:NS  PSYRAT AH attribution  Post: d=0.41*, p:NS; 36 weeks follow-up: d=0.2*, p:NS  PSYRAT AH frequency  Post: d=0.74*, p:NS; 36 weeks follow-up: d=1.115*, p:NS  PSYRAT AH loudness  Post: d=0.54*, p:NS; 36 weeks follow-up: d=1.049*, p:NS  ***Severity of other symptoms***  HADS anxiety  Post: d=1.069*, p:NS; 36 weeks follow-up: d=1.025*, p:NS  HADS depression  Post: d=0.758*, p:NS; 36 weeks follow-up: d=1.053*, p:NS  ***Relation with voices***  VAY voice dominance  Post: d=0.778*, p:NS; 36 weeks follow-up: d=0.824*, p=NS  VAY voice intrusiveness  Post: d=0.391*, p=NR; 36 weeks follow-up: d=0.729*, p=NS  VAY hearer dependence  Post: d=0.362*, p=NS; 36 weeks follow-up: d=0.659*, p=NS  VAY hearer distance  Post: d=0.333*, p:NS; 36 weeks follow-up: d=0.683*, p:NS  Between group  ***Severity of AH***  PSYRAT AH total  **Post: d=1.4, CI= -12.9; -1.3;** 36 weeks follow-up: NS  PSYRAT AH distress  **Post: d=1.3 CI= -7.5; -0.9; 36 weeks follow-up: d=1.4, CI= -8.7; -0.2**  PSYRAT AH attribution  **Post: d=0.8 CI= -2.9; -0.1;** 36 weeks follow-up: NS  PSYRAT AH frequency  Post: NS; 36 weeks follow-up: NS  PSYRAT AH loudness  Post: NS; 36 weeks follow-up: NS  ***Severity of other symptoms***  HADS anxiety  Post: NS; 36 weeks follow-up: NS  HADS depression  **Post: d=0.7, CI= -6.8; -1.3;** 36 weeks follow-up: NS  ***Relation with voices***  VAY voice dominance  Post: NS; 36 weeks follow-up: NS  VAY voice intrusiveness  Post: NS; 36 weeks follow-up: NS  VAY hearer dependence  Post: NS; 36 weeks follow-up: NS  VAY hearer distance  Post: NS; 36 weeks follow-up: NS | Moderate |
| *Avatar therapy / Virtual reality assisted therapy* | | | | | | | |
| Dellazzizo et al., 2018 | 1 | Treatment-resistant SCZ | Case repot | None | 7 | ***Severity of AH***  Frequency (qualitative measure)  Without changing any medication throughout the therapy, voices diminished by 80–90%. Instead of hearing voices 10 to 15 times per day, they were only present one to three times per day.  ***Belief about voices***  Belief about voices (qualitative measure)  Instead of avoiding contact and suffering from the voice’s intrusions, they learned how to confront the voice. As this occurred, they began to perceive the voice as less powerful during the final sessions. Throughout AT, they learned how to take control, confront, and assert themselves. | Very low |
| Leff et al., 2013 | 26  (16 AT,  12 TAU) | Antipsychotic medication resistant irrespective of diagnosis | Single blind randomised proof of concept study | TAU | 6 | Within group immediate  ***Severity of AH***  PSYRAT AH total  **Post: d=0.846*, p=0<0.029; 3 months follow-up: d=1.446*, p<0.001**  ***Belief about voices***  BAVQ-R total  Post: p=NS; **3 months follow-up: d=0.850*, p=0.014**  ***Severity of other symptoms***  CDS  Post: NS; **3 months follow-up: d=0.895*, p=0.036**  Within group delayed  ***Severity of AH***  PSYRAT AH total  **Post: d=1.090*, p:**; 3 months follow-up: d=1.609, p:****  ***Belief about voices***  BAVQ-R total  Post: NS; **3 months follow-up: d=0.727, p:****  ***Severity of other symptoms***  CDS  Post: NS; **3 months follow-up: d=0.884, p:****  ****NS between immediate and delayed group** | Low-moderate |
| de Sert et al., 2018 | 15  (15 VRT,  7 TAU) | Treatment-resistant SCZ | Partial cross-over trial pilot study | TAU | 7 | Within group  ***Severity of AH***  PSYRAT AH total  **Post: d=1.12, p<0.01; 3 months follow-up: d=1.20, p<0.01**  PSYRAT AH distress  **Post: d=1.33, p<0.001; 3 months follow-up: d=1.31, p<0.001**  PSYRAT AH frequency  Post: NS; 3 months follow-up: NS  PSYRAT AH attribution  Post: NS; **3 months follow-up: d=0.91*, p<0.05**  PSYRATAH loudness  Post: NS; 3 months follow-up: NS  ***Belief about voices***  BAVQ-R total  **Post: d=0.98, p<0.05; 3 months follow-up: d=0.87, p<0.013**  BAVQ-R malevolence  **Post: d=0.58, p<0.05; 3 months follow-up: d=0.70, p<0.05**  BAVQ-R omnipotence  **Post: d=0.91, p<0.05; 3 months follow-up: d=0.59, p<0.05**  ***Severity of other symptoms***  PANSS total  Post: NS; **3 months follow-up: d=0.78, p<0.05**  PANSS positive  Post: NS; 3 months follow-up: NS  PANSS negative  Post: NS; 3 months follow-up: NS  PANSS general  **Post: d=0.61, p<0.05; 3 months follow-up: d=1.13, p<0.01**  BDI  **Post: d=0.586, p<0.05; 3 months follow-up: d=0.82, p<0.05**  ****NS between immediate and delayed group** | Low-moderate |
| Craig et al., 2018 | 150  (75 AT,  75 SC) | SCZ, SZA bipolar disorder (7), Unspecific psychosis (8), depression with psychotic symptoms (4) | Single blind RCT | SC | 6 | Within group  ***Severity of AH***  PSYRAT AH total  Post: d=0.830*, p:NR; 24 weeks follow-up: d=0.872*, p:NR  PSYRAT AH distress  Post: d=0.856*, p:NR; 24 weeks follow-up: d=0.967*, p:NR  PSYRAT AH frequency  Post: d=0.729*, p:NR; 24 weeks follow-up: d=0.675*, p:NR  VPDS total  Post: d=0.523*, p:NR; 24 weeks follow-up: d=0.576*, p:NR  VPDS voice power  Post: d=0.355*, p:NR; 24 weeks follow-up: d=0.313*, p:NR  VPDS assertiveness  Post: d=0.406*, p:NR; 24 weeks follow-up: d=0.406*, p:NR  VAAS acceptance  Post: d=0.666*, p:NR; 24 weeks follow-up: d=0.659*, p:NR  VAAS action  Post: d=0.479*, p:NR; 24 weeks follow-up: d=0.532*, p:NR  ***Belief about voices***  BAVQ-R total  Post: d=0.470*, p:NR; 24 weeks follow-up: d=0.611*, p:NR  BAVQ-R malevolence  Post: d=0.472*, p:NR; 24 weeks follow-up: d=0.513*, p:NR  BAVQ-R benevolence  Post: NS; 24 weeks follow-up: NS  BAVQ-R omnipotence  Post: d=0.535*, p:NR; 24 weeks follow-up: d=0.577*, p:NR  ***Severity of other symptoms***  SAPS  Post: d=0.286*, p:NR; 24 weeks follow-up: d=0.279*, p:NR  SANS  Post: NS; 24 weeks follow-up: NS  Calgary depression scale  Post: d=0.444*, p:NR; 24 weeks follow-up: d=0.344*, p:NR  DASS-21 total  Post: d=0.382*, p:NR; 24 weeks follow-up: d=0.355*, p:NR  DASS-21 anxiety  Post: d=0.390*, p:NR; 24 weeks follow-up: d=0.287*, p:NR  DASS-21 stress  Post: d=0.342*, p:NR; 24 weeks follow-up: d=0.417*, p:NR  DASS-21 depression  Post: d=0.312*, p:NR; 24 weeks follow-up: d=0.275*, p:NR  ***Self-esteem***  RSES  Post: d=0.382*, p:NR; 24 weeks follow-up: d=0.294*, p:NR  Between group  ***Severity of AH***  PSYRAT AH total  **Post: d=0.8, p=0·009;** 24 weeks follow-up: NS  PSYRAT AH distress  **Post: d=0.505*, p=0.017;** 24 weeks follow-up: NS  PSYRAT AH frequency  **Post: d=0.595*, p=0.0013**; 24 weeks follow-up: NS  VPDS total  **Post: d=0.379*, p=0.026;** 24 weeks follow-up: maintained  VPDS voice power  Post: NS; 24 weeks follow-up: NS  VPDS assertiveness  **Post: d=0.533*, p=0.084;** 24 weeks follow-up: maintained  VAAS acceptance  **Post: d=0.502*, p=0.033;** 24 weeks follow-up: maintained  VAAS action  **Post: d=0.441*, p=0.019;** 24 weeks follow-up: maintained  ***Belief about voices***  BAVQ-R total  **Post: d=0.497*, p=0.018;** 24 weeks follow-up: maintained  BAVQ-R malevolence  Post: NS; 24 weeks follow-up: NS  BAVQ-R benevolence  Post: NS; 24 weeks follow-up: NS  BAVQ-R omnipotence  **Post: d=0.501*, p=0.038;** 24 weeks follow-up: NS  ***Severity of other symptoms***  SAPS  Post: NS; 24 weeks follow-up: NS  SANS  Post: NS; 24 weeks follow-up: NS  Calgary depression scale  Post: NS; 24 weeks follow-up: NS  DASS-21 total  Post: NS; 24 weeks follow-up: NS  DASS-21 anxiety  Post: NS; 24 weeks follow-up: NS  DASS-21 stress  Post: NS; 24 weeks follow-up: NS  DASS-21 depression  Post: NS; 24 weeks follow-up: NS  ***Self-esteem***  RSES  Post: NS; 24 weeks follow-up: NS | High |
| Dellazizzo et al., 2021 | 74  (37 VRT,  37 CBT) | Treatment-resistant SCZ and SZA | Nonblind randomized trial | CBT | 9 | Within group  ***Severity of AH***  PSYRAT AH total  **3 months follow-up: d=1.080, p<0.001; 1-year follow-up: maintained**  PSYRAT AH distress  **3 months follow-up: d=0.998, p<0.001 1-year follow-up: maintained**  PSYRAT AH frequency  **3 months follow-up: d=0.701, p=0.021; 1-year follow-up: maintained**  PSYRAT AH attribution  **3 months follow-up: d=0.665, p=0.004; 1-year follow-up: maintained**  PSYRAT AH loudness  3 months follow-up: NS; 1-year follow-up: NS  ***Belief about voices***  BAVQ-R total  3 months follow-up: NS; 1-year follow-up: NS  BAVQ-R persecutory beliefs  **3 months follow-up:d=0.438, p=0.039;** 1-year follow-up: NS  BAVQ-R benevolence  3 months follow-up: NS; 1-year follow-up: NS  BAVQ-R engagement  3 months follow-up: NS; 1-year follow-up: NS  BAVQ-R resistance  3 months follow-up: NS; 1-year follow-up: NS  ***Severity of other symptoms***  PANSS total  **3 months follow-up: d=0.651, p=0.015;** **1-year follow-up:** **maintained**  PANSS positive  3 months follow-up: NS; 1-year follow-up: NS  PANSS negative  3 months follow-up: NS; 1-year follow-up: NS  PANSS disorganized  3 months follow-up: NS; 1-year follow-up: NS  PANSS anxio-depressive  **3 months follow-up: d=0.786, p<0.001;** **1-year follow-up:** **maintained**  PANSS excited/hostility  **3 months follow-up: d=0.724, p=0.004;** **1-year follow-up:** **maintained**  BDI-II total  **3 months follow-up: d=0.577, p=0.004;** **1-year follow-up:** **maintained**  BDI-II cognitive  **3 months follow-up: d=0.590, p=0.001;** **1-year follow-up:** **maintained**  BDI-II somatic-affective  3 months follow-up: NS; 1-year follow-up: NS  ***Quality of life***  QLESQ-SF  **Post: 3-months follow-up: d=0.637, p=0.001;** **1-year follow-up:** **maintained**  Between group  ***Severity of AH***  PSYRAT AH total  months follow-up: NS 1-year follow-up: NS  PSYRAT AH distress  3 months follow-up: NS 1-year follow-up: NS  PSYRAT AH frequency  3 months follow-up: NS 1-year follow-up: NS  PSYRAT AH attribution  3 months follow-up: NS 1-year follow-up: NS  PSYRAT AH loudness  3 months follow-up: NS 1-year follow-up: NS  ***Belief about voices***  BAVQ-R total  3 months follow-up: NS 1-year follow-up: NS  BAVQ-R persecutory beliefs  3 months follow-up: NS 1-year follow-up: NS  BAVQ-R benevolence  3 months follow-up: NS 1-year follow-up: NS  BAVQ-R engagement  3 months follow-up: NS 1-year follow-up: NS  BAVQ-R resistance  3 months follow-up: NS 1-year follow-up: NS  ***Severity of other symptoms***  PANSS total  3 months follow-up: NS 1-year follow-up: NS  PANSS positive  3 months follow-up: NS 1-year follow-up: NS  PANSS negative  3 months follow-up: NS 1-year follow-up: NS  PANSS disorganized  3 months follow-up: NS 1-year follow-up: NS  PANSS anxio-depressive  **Overall: p=0.025**  PANSS excited/hostility  3 months follow-up: NS 1-year follow-up: NS  BDI-II total  3 months follow-up: NS 1-year follow-up: NS  BDI-II cognitive  3 months follow-up: NS 1-year follow-up: NS  BDI-II somatic-affective  3 months follow-up: NS 1-year follow-up: NS | Moderate |
| *Avatar therapy / Virtual reality-assisted therapy variants* | | | | | | | |
| Cichocki et al., 2016 | 1 | SCZ | Case report | None | 5 | ***Severity of AH***  Frequency qualitative  If some auditory hallucinations do occur, they are negligible and have no significant disruptive effect on the patient’s social functioning.  ***Severity of other symptoms***  Positive symptoms qualitative  As the psychotic symptoms had desisted, the dosage of anti-psychotic drugs could be considerably reduced. He remained essentially in remission from his positive symptoms. | Very low |
| Dellazizzo et al., 2018 | 1 | Ultra treatment-resistant SCZ | Case report | None | 7 | ***Severity of AH***  Frequency qualitative  While the voice work in therapy was gone, unfortunately the PSYRATS was unable to capture this clinical amelioration as Mr. Smith still had several voices.  ***Severity of other symptoms***  PANSS total  Post CBT: Post VRT: ↓ 24%; 3 months follow-up: remained stable  PANSS positive  Post CBT: Post VRT: ↓ 27%; 3 months follow-up: remained stable  PANSS negative  Post CBT: Post VRT: reduction of 29%; 3 months follow-up: remained stable  PANSS general  Post CBT: Post VRT: reduction of 20%; 3 months follow-up: remained stable  BDI-II  Post CBT: Post VRT: reduction of 75% | Very low |
| Dellazizzo et al., 2020 | 10 | Treatment-resistant SCZ and SZA | Nonblind nonrandomised proof of concept | None | 9 | ***Severity of AH***  PSYRAT AH total  **Post: d= 0.838; p=0.026 3 months follow-up: d=1.043, p=0.001**  PSYRAT AH distress  Post: NS; **3 months follow-up: d=0.898, p=0.007**  PSYRAT AH frequency  **Post: d= 0.714, p= 0.022; 3 months follow-up: d=0.859, p=0.001**  PSYRAT AH attribution  **Post: d= 0.889, p= 0.015; 3 months follow-up: d=1.020, p=0.001**  PSYRAT AH loudness  Post: NS; **3 months follow-up: d= 0.946, p= 0.050**  ***Belief about voices***  BAVQ-R total  **Post: d=0.461, p=0.037;** 3 months follow-up: NS  BAVQ-R persecutory beliefs  **Post: d= 0.555, p=0.049;** 3 months follow-up: NS  BAVQ-R benevolence  Post: NS; 3 months follow-up: NS  BAVQ-R engagement  Post: NS; 3 months follow-up: NS  BAVQ-R resistance  Post: NS; 3 months follow-up: NS  ***Severity of other symptoms***  PANSS total  **Post: d=0.953, p=0.003**; 3 months follow-up: NS  PANSS positive  **Post: d= 1.128, p=0.007;** 3 months follow-up: NS  PANSS negative  **Post: d=0.558, p= 0.044;** 3 months follow-up: NS  PANSS disorganized  **Post: d= 0.545, p= 0.032; 3 months follow-up: d=1.040, p=0.013**  PANSS anxio-depressive/affective  Post: NS; 3 months follow-up: NS  PANSS excited/hostility  **Post: d= 0.955, p=0.016;** 3 months follow-up: NS  BDI-11 total  **Post: d= 0.783, p= 0.004; 3 months follow-up: d=1.020, p<0.001** | Low |
| Stefaniak et al., 2017 | 1 | Treatment-resistant SCZ | Case report | None | 20 | ***Severity of AH***  Frequency qualitative  Post: A new way of thinking about voices was strengthened. The patient was saying: “most of the time, I do not hear voices. It happened that I felt very well for the first time in many months. I had the feeling that I regained sight and the ability to feel emotions. 6 months follow-up: improvement involving reduced frequency of hallucinations and being more successful in coping with them in the periods of aggravation.  Intrusiveness of voice qualitative  Post and 6 months follow-up: a significant reduction in the frequency and intrusiveness of voices and sustainability of these effects | Very low |
| Stefaniak et al, 2019 | 23  (13 AT,  10 TAU) | Schizophrenia (20)  Not reported (3) | Nonblind randomised pilot study | TAU | 8 | Within group  ***Severity of AH***  PSYRAT AH total  **Post: d=1.486*, p<0.0001; 3 months follow-up: d=1.837*, p= 0.0131**  VPD total  **Post: d=1.338*, p<0.0001;** 3 months follow-up: NS | Low |

* Effect sizes and/or % changes were calculated When not obtained within the corresponding studies.

AH : auditory hallucination; AT : Avatar therapy; BAVQ-R : Beliefs About Voices Questionnaire-Revised; BDI : Beck Depression Inventory; BPD : borderline personality disorder; BPRS-E : Brief Psychiatric Rating Scale - Expanded Version; BPRS : Brief Psychiatric Rating Scale; CBT : cognitive behavioral therapy; CDS : Calgary depression scale; d : Cohen’s d; DASS : Depression Anxiety and Stress Scale; EFC : Experience focused Counselling; HADS : Hospital Anxiety and Depression Scale; NOS : not otherwise specified; NR : not reported; NS : not significant; p : p-value; PANSS : Positive And Negative Syndrome Scale; Post : post treatment; PSYRAT : Psychotic Symptom Rating Scales; RCT : Randomized Controlled Trial; RSES : Rosenberg Self-Esteem Scale; RT : Relating therapy; SANS: Scale for assessment of negative symptoms; SAPS: Scale for assessment of positive symptoms; SC : supportive counselling; SCZ :schizophrenia; SZA : schizoaffective disorder; TAU : treatment as usual; VAAS : Voices Acceptance and Action Scale; VAY : Voice and You; VRT : Virtual reality-assisted therapy; WEMWBS : Warwick Edinburgh Mental Wellbeing Scale.

**Table S3.** PRISMA Checklist.

| **Section/topic** | **#** | **Checklist item** | **Reported on page #** |
| --- | --- | --- | --- |
| **TITLE** | | |  |
| Title | 1 | Identify the report as a systematic review, meta-analysis, or both. | 1 |
| **ABSTRACT** | | |  |
| Structured summary | 2 | Provide a structured summary including, as applicable: background; objectives; data sources; study eligibility criteria, participants, and interventions; study appraisal and synthesis methods; results; limitations; conclusions and implications of key findings; systematic review registration number. | 2 |
| **INTRODUCTION** | | |  |
| Rationale | 3 | Describe the rationale for the review in the context of what is already known. | 3-4 |
| Objectives | 4 | Provide an explicit statement of questions being addressed with reference to participants, interventions, comparisons, outcomes, and study design (PICOS). | 4 |
| **METHODS** | | |  |
| Protocol and registration | 5 | Indicate if a review protocol exists, if and where it can be accessed (e.g., Web address), and, if available, provide registration information including registration number. | NA |
| Eligibility criteria | 6 | Specify study characteristics (e.g., PICOS, length of follow-up) and report characteristics (e.g., years considered, language, publication status) used as criteria for eligibility, giving rationale. | 5-6 |
| Information sources | 7 | Describe all information sources (e.g., databases with dates of coverage, contact with study authors to identify additional studies) in the search and date last searched. | 5-6 |
| Search | 8 | Present full electronic search strategy for at least one database, including any limits used, such that it could be repeated. | Suppl. material |
| Study selection | 9 | State the process for selecting studies (i.e., screening, eligibility, included in systematic review, and, if applicable, included in the meta-analysis). | 5-6 |
| Data collection process | 10 | Describe method of data extraction from reports (e.g., piloted forms, independently, in duplicate) and any processes for obtaining and confirming data from investigators. | 5-6 |
| Data items | 11 | List and define all variables for which data were sought (e.g., PICOS, funding sources) and any assumptions and simplifications made. | 5-6 |

| **Section/topic** | **#** | **Checklist item** | **Reported on page #** |
| --- | --- | --- | --- |
| Risk of bias in individual studies | 12 | Describe methods used for assessing risk of bias of individual studies (including specification of whether this was done at the study or outcome level), and how this information is to be used in any data synthesis. | NA |
| Summary measures | 13 | State the principal summary measures (e.g., risk ratio, difference in means). | 5-6 |
| Synthesis of results | 14 | Describe the methods of handling data and combining results of studies, if done, including measures of consistency (e.g., I^2^) for each meta-analysis. | NA |
| Risk of bias across studies | 15 | Specify any assessment of risk of bias that may affect the cumulative evidence (e.g., publication bias, selective reporting within studies). | NA |
| Additional analyses | 16 | Describe methods of additional analyses (e.g., sensitivity or subgroup analyses, meta-regression), if done, indicating which were pre-specified. | NA |
| **RESULTS** | | |  |
| Study selection | 17 | Give numbers of studies screened, assessed for eligibility, and included in the review, with reasons for exclusions at each stage, ideally with a flow diagram. | 8 |
| Study characteristics | 18 | For each study, present characteristics for which data were extracted (e.g., study size, PICOS, follow-up period) and provide the citations. | 8-18 |
| Risk of bias within studies | 19 | Present data on risk of bias of each study and, if available, any outcome level assessment (see item 12). | 8-18 |
| Results of individual studies | 20 | For all outcomes considered (benefits or harms), present, for each study: (a) simple summary data for each intervention group (b) effect estimates and confidence intervals, ideally with a forest plot. | 8-18 |
| Synthesis of results | 21 | Present results of each meta-analysis done, including confidence intervals and measures of consistency. | Suppl. material |
| Risk of bias across studies | 22 | Present results of any assessment of risk of bias across studies (see Item 15). | NA |
| Additional analysis | 23 | Give results of additional analyses, if done (e.g., sensitivity or subgroup analyses, meta-regression [see Item 16]). | NA |
| **DISCUSSION** | | |  |
| Summary of evidence | 24 | Summarize the main findings including the strength of evidence for each main outcome; consider their relevance to key groups (e.g., healthcare providers, users, and policy makers). | 19-22 |
| Limitations | 25 | Discuss limitations at study and outcome level (e.g., risk of bias), and at review-level (e.g., incomplete retrieval of identified research, reporting bias). | 21 |
| Conclusions | 26 | Provide a general interpretation of the results in the context of other evidence, and implications for future research. | 22 |
| **FUNDING** | | |  |
| Funding | 27 | Describe sources of funding for the systematic review and other support (e.g., supply of data); role of funders for the systematic review. | 22 |

*From:*  Moher D, Liberati A, Tetzlaff J, Altman DG, The PRISMA Group (2009). Preferred Reporting Items for Systematic Reviews and Meta-Analyses: The PRISMA Statement. PLoS Med 6(7): e1000097. doi:10.1371/journal.pmed1000097

For more information, visit: **www.prisma-statement.org**.
